# Solvent Exclusion Effect on Infrared Absorption Spectroscopy

**DOI:** 10.1021/acs.analchem.5c02700

**Published:** 2025-07-09

**Authors:** Young Jong Lee, Seong-Min Kim, Sang Hak Lee, Charles H. Camp, Bonghwan Chon

**Affiliations:** † Biosystems and Biomaterials Division, 10833 National Institute of Standards and Technology, Gaithersburg, Maryland 20899, United States; ‡ Department of Chemistry, 34996 Pusan National University, Busan 46241, Korea

## Abstract

Absorption spectroscopy of a solution containing an analyte solute typically uses the transmission through a solvent as a reference based on the assumption that the solvent does not absorb light. However, when a solvent absorbs light, the resulting absorbance becomes lower and often negative because of the reduced number of solvent molecules due to the space taken by solute molecules. The solvent exclusion (SE) effect is problematic for accurate quantitation and analysis across various absorption spectroscopies, especially near the broad, strong water absorption peaks. The commonly practiced method of subtracting a scaled reference water spectrum seems ideal; however, this requires extensively broad spectra that are not available with the combination of flow cell chamber thickness and currently available high-intensity laser sources. In this work, we present a simple volumetric SE correction method using partial specific volume (PSV) to successfully retrieve the solute-only absorption spectrum of globular proteins in aqueous solutions. We demonstrate this method with spectra acquired with an in-house-developed, state-of-the-art quantum cascade laser (QCL) spectroscopy system with solvent absorption compensation (SAC) for unprecedented molecular sensitivity. Additionally, we discuss the effectiveness of this simplified PSV-based SE correction for globular solutes and small-molecule solutes using a model consisting of a solvation shell and a solute core.

## Graphical abstract

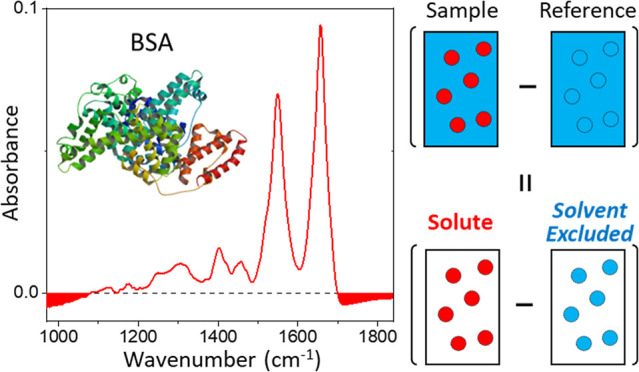

## Introduction

The absorbance of an analyte in a sample solution is expressed as *A* ≡ −log_10_(*I*_sample_/*I*_reference_), where *I*_sample_ and *I*_reference_ are the transmitted light intensities through a sample solution and a reference solvent, respectively. This method works effectively when a solvent is transparent and an analyte absorbs light exclusively. However, when the absorption cross section of a reference solvent is non-negligible compared to that of an analyte, the apparent absorbance spectrum calculated by the above Beer’s law deviates from the true absorbance spectrum of the analyte. One reason for the discrepancy is the solvent exclusion (SE) by solute molecules. When a solute is dissolved into a solution, the solute molecules will occupy space and exclude solvent molecules, reducing the solvent concentration. The apparent absorption will thus decrease due to the reduced absorption by excluded solvent molecules though the reference sample remained unchanged. Thermodynamically, the excluded volume of a solvent can be represented by the partial specific volume (PSV) of a solute. PSV refers to the change in the volume of a solution by a solute, expressed in the unit of mL/g. PSV is a hydrodynamic parameter used in data analysis of solution-state experiments, such as analytical ultracentrifugation and small-angle X-ray or neutron scattering.^1^

The effect of SE and its volumetric correction methods has been considered in various absorption spectroscopies, such as near-infrared,^2,3^ terahertz,^4^ and Fourier-transform IR (FT-IR) absorption spectroscopy.^5–8^ However, strong absorption by solvent molecules often requires a short path length of a sample cell for a sufficient signal from the detection system. For example, FT-IR spectroscopy allows sample cells with a path length of <10 μm for typical benchtop IR light sources, e.g., Globar.^6^ This short path length, incompatible with typical microfluidic sample introduction due to pressure build-up, requires a detachable cell for loading a sample and a reference. In this sample loading scheme, the variation in optical path length during transmission measurements becomes unavoidable, and its consequence is greater than the SE effect. Thus, instead of using PSV, an absorption spectrum is calculated by multiplying a proportional coefficient to the reference absorption spectrum iteratively until apparent absorbance becomes zero in a quiescent frequency region of organic solutes.^5,6,8,9^ However, because this iterative approach requires a sufficiently broad frequency range covering a quiescent region, it cannot be used for a spectroscopy system with a relatively narrow frequency range, such as quantum cascade laser (QCL)-based IR spectrometers.

QCL provides high photon flux over relatively narrow spectral windows within the mid-IR region. This has enabled significantly enhanced detection of analytes in aqueous environments in longer path length sample chambers, and even affords the use of microfluidics for sample exchange and flow, which further improves quantitative analysis by minimizing thickness variation during measurements. Recently, QCL-based IR spectroscopy demonstrated pathlengths of up to 38 μm,^10^ and typically 25 μm,^11^ even across the strong water bending absorption region around 1640 cm^–1^. Furthermore, we have developed a new optical technique, solvent absorption compensation (SAC),^11^ that improves the signal-to-noise ratio more than a hundred times over direct absorption measurements. Upgraded with a double-beam modulation technique^12^ and a broadband light control method,^13^ the SAC-IR spectroscopy became more sensitive over the entire QCL scanning range. The currently available QCL-IR frequency range (950–1840 cm^–1^)^13^ covers most of the fingerprint peaks of organic molecules but is not sufficiently broad to cover the frequency ranges previously described for FT-IR SE correction.^5,6,8,9^

In this paper, we examine the SE absorbance correction by calculating the volume of water excluded by a solute with its reported PSV value. We derive the formula based on a noninteracting solute model and test it with the absorption spectra of a few globular proteins. We report the SE effect on the absorption spectra of small-molecule solutes and discuss why the simple volumetric correction model cannot correct the SE effect. We discuss the relatively significant contribution of the strong interaction with solute molecules to the solvent spectrum.

## Experimental Section

A detailed description of the SAC-IR spectroscopy system can be found in our earlier papers.^11–13^ Briefly, a QCL generated monochromatic light (∼1 cm^–1^ line width) scanning from 970 cm^–1^ to 1840 cm^–1^. In this work, the laser frequency was scanned with a constant speed of 2000 cm^–1^/s. The laser output was split into a sample beam and a reference beam, and the intensities of the two beams were independently adjusted by the double-pass acousto-optic modulator units^13^ for the double-beam modulation.^12^ The two beams were sent toward a sample cell and a reference cell separately and focused by an off-axis parabolic mirror onto a single liquid nitrogen-cooled mercury–cadmium-telluride (MCT) detector with a signal processed by a lock-in amplifier. While wavelength scanning, SAC-programmed voltages were applied to the analog input ports of the two AOM drivers separately. The reference cell, consisting of two CaF_2_ windows and a spacer with a nominal path length of 26 μm, was filled with distilled water. The sample cell also consisted of two CaF_2_ windows and a spacer with a nominal path length of 26 μm. A programmable perfusion system under constant pressure injected a sample solution and a reference solvent alternatively through the sample cell. The entire spectroscopy system was enclosed and purged with dry air during measurement.

We used a SAC-based IR microscopy system to acquire the IR spectrum of a poly­(methyl methacrylate) (PMMA) particle surrounded by water. The details of the SAC-IR microscopy system can be found elsewhere.^14^ Briefly, a QCL tunable from 900 cm^–1^ to 1774 cm^–1^ was used as an IR source. For the PMMA bead sample, dispersion water was dropped on the 5 μm PMMA beads softly attached to a 1 mm thick CaF_2_ slide and sandwiched with a 25 μm spacer and another 1 mm thick CaF_2_ slide. The beam was focused onto the sample by a reflective objective lens. The transmitted light through the sample was collimated by a refractive objective lens and then focused onto a liquid N_2_-cooled MCT detector by an off-axis parabolic mirror.

Powder samples of bovine serum albumin (BSA), β-lactoglobulin (β-LG), spermine, and mannose were used as received from the manufacturers without drying. Sample solutions were prepared at concentrations close to 10 mg/mL by weighing the powder samples. The concentrations of BSA and β-LG solutions were calibrated with separately measured UV absorbances at 280 nm. The concentrations of mannose, galactose, and spermine solutions were calculated from the powder weights without further calibration. The pH of the spermine solution was adjusted to close to 7 by adding HCl solution.

## Results

### Solvent Exclusion (SE) Effect on Apparent Absorbance

In an absorption measurement, the transmission spectrum of a solution sample (*I* ^s^) and the transmission spectrum of a reference (*I* ^r^) are converted into absorbance using Beer’s law with the assumption that the differences in reflection and scattering are negligible

1 $$A^{apparent}\equiv -\log_{10}\left(\frac{I^{s}}{I^{r}}\right)$$

where *A* ^apparent^ is apparent absorbance. Figure 1 shows the *A* ^apparent^ spectra acquired from three different solutions and one polymer bead sample immersed in water. In Figure 1a, a solution containing BSA, a globular protein with a 66 kDa molar mass, exhibits representative peptide absorption peaks, such as the amide I band at 1650 cm^–1^ and the amide II band at 1550 cm^–1^. The spectrum also shows negative *A* ^apparent^ at the frequency range lower than 1100 cm^–1^ and higher than 1700 cm^–1^. The absorption *A* ^apparent^ of mannose in Figure 1b also shows negative absorbance. The strong peak between 1000 cm^–1^ and 1200 cm^–1^ corresponds to the 6-membered monosaccharide ring band, while a substantial negative dip is observed at higher frequencies than 1500 cm^–1^. The spectrum of a spermine solution in Figure 1c shows a significant lowering over the entire frequency range with a deeper dip around 1650 cm^–1^. In Figure 1d, the absorption spectrum of a single PMMA bead immersed in water also exhibits a deep negative absorbance near 1640 cm^–1^ near the strong ester carbonyl peak at 1720 cm^–1^. The negative absorbances appearing in the apparent absorption spectra in Figure 1 indicate that the sample solutions absorb less light than the reference water.

**Figure 1:**
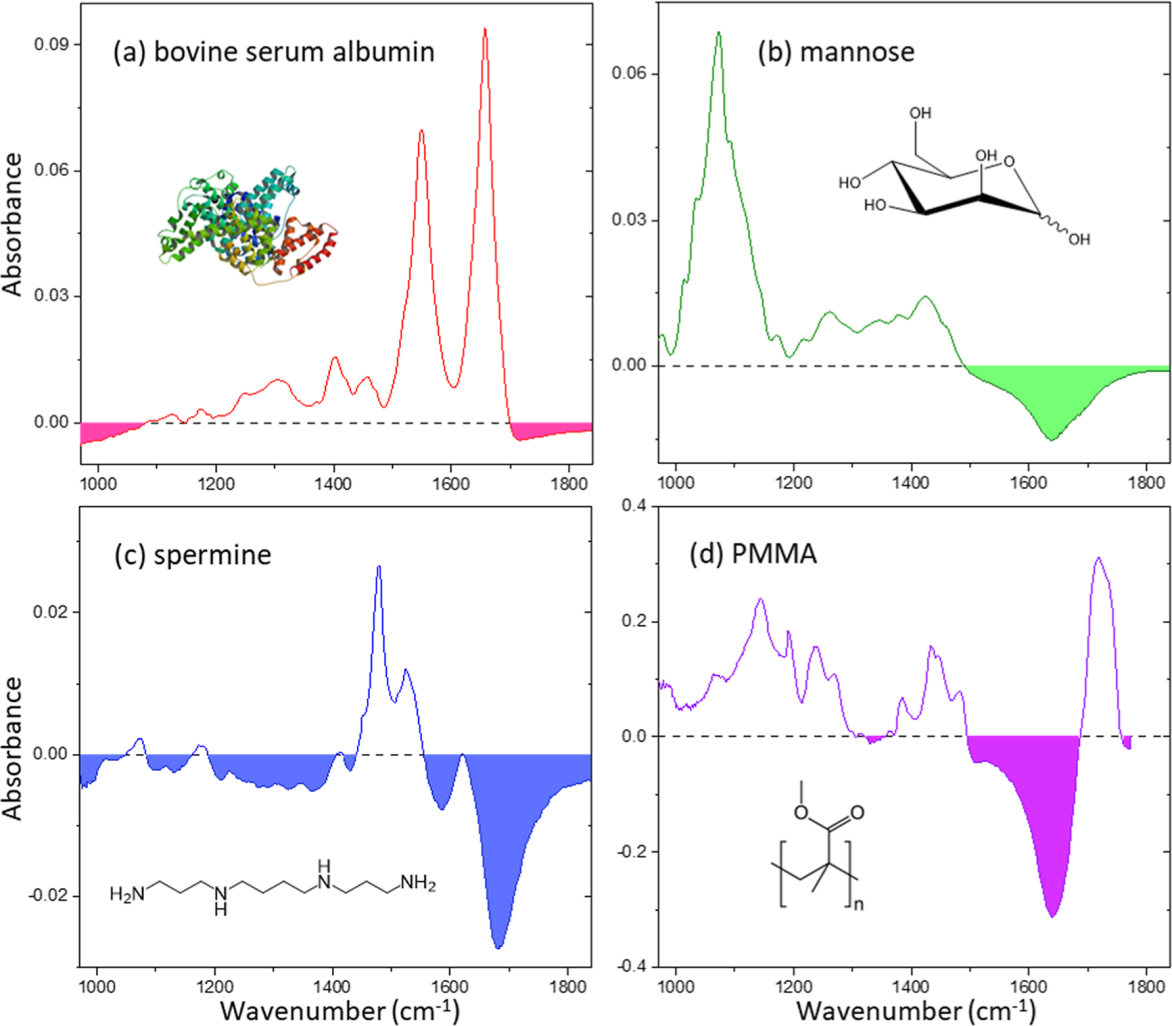
Absorption spectra of various aqueous solution samples calculated with neat water as a reference: (a) bovine serum albumin solution (10 mg/mL), (b) mannose solution (10 mg/mL), (c) spermine solution (10 mg/mL), and (d) 5 μm diameter PMMA bead immersed in water. The shaded regions highlight negative absorbance.

To discuss the lowering of *A* ^apparent^ observed in aqueous solution samples, we rewrite eq 1 as $A^{apparent}=-\log_{10}(\frac{I^{s}}{I^{empty}})+\log_{10}(\frac{I^{r}}{I^{empty}})=A^{s}-A^{r}$ , where *A* ^s^ and *A* ^r^ are the absorbances of a sample solution and a reference solvent, respectively, with respect to *I* ^empty^, which need not to be measured experimentally. For clarity, this manuscript will refer to a “reference” solution as a “solvent,” although the reference solution may contain not only neat solvent but also other excipients. Similarly, a “solute” indicates an analyte of interest. For simplicity, we assume a noninteracting solute model, where the solvent spectrum solely depends on the number of solvent molecules. Then, we express *A* ^s^ as the sum of the absorbances of a solute (*A*_solute_) and a solvent (*A*_solv_^s^). Also, *A* ^r^ is replaced with *A*_solv_^r^. Then, we can rearrange *A* ^apparent^ in eq 1 as

2 $$A^{apparent}=A^{s}-A^{r}=\left(A_{solute}+A_{solv}^{s}\right)-A_{solv}^{r}=A_{solute}-\left(A_{solv}^{r}-A_{solv}^{s}\right)$$

Figure 2a illustrates the relation of *A* ^apparent^ with *A*_solute_, *A*_solv_^s^, and *A*_solv_^r^. The solute molecules (the red circles) excluded the equivalent volume of solvent molecules (the blue circles), corresponding to the reduced number of solvent molecules in the measurement beam path. The absorbance reduction due to a reduced number of solvent molecules can be expressed as *A*_solv_^excl^ = (*A*_solv_^r^ – *A*_solv_^s^), and then, eq 2 will become

3 $$A^{apparent}=A_{solute}-A_{solv}^{excl}$$

**Figure 2:**
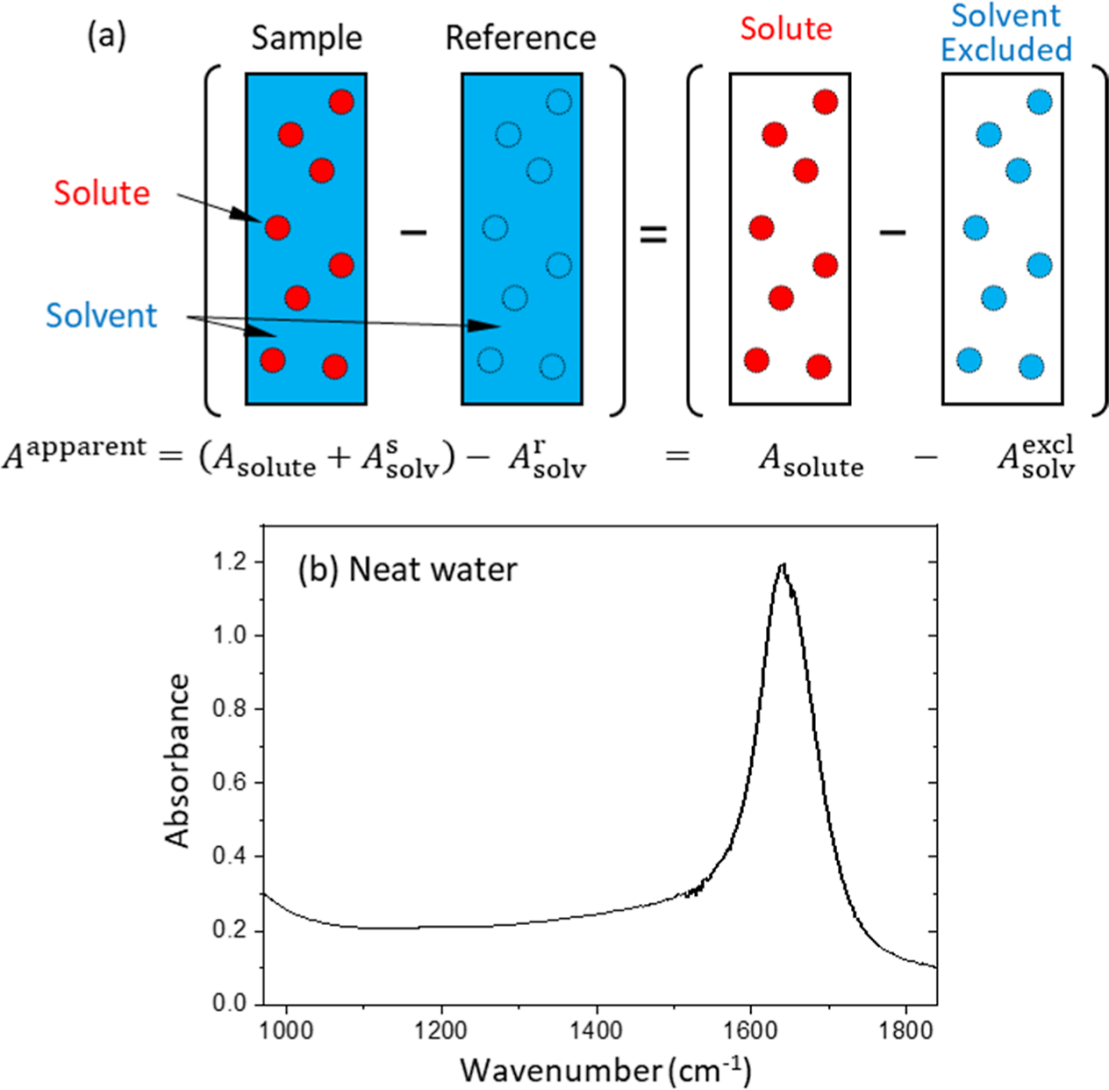
(a) Illustration of the solvent exclusion (SE) effect on an apparent absorbance (*A* ^apparent^), corresponding to the absorbance difference between a sample solution cell and a reference solvent cell. The sample solution consists of an analyte solute (red) and a solvent (blue), whereas the reference cell contains the solvent exclusively. (b) Absorption spectrum of neat water of 10 μm path length (see the details in Supporting Information).

When the absorption cross section of a solvent is nonzero, the SE effect will make *A*_solv_^excl^ > 0, and then, *A* ^apparent^ < *A*_solute_, resulting in the lowering of the absorption spectra of Figure 1. The SE effect is also consistent with the distinct absorption dip observed near 1650 cm^–1^ in Figure 1b–d, where the water bending mode is located, as shown in Figure 2b.

### Partial Specific Volume (PSV) and SE

PSV represents the solution volume change by the added mass of a solute. PSV of the *i*-th solute, denoted as ν_*i*_, is defined as the partial derivative of the solution volume, *V*, with respect to the solute mass, *m*_*i*_

4 $$\nu_{i}\equiv {\left(\frac{\partial V}{\partial m_{i}}\right)}_{T,P,m_{j\neq i}}\approx {\left(\frac{\Delta V}{m_{i}}\right)}_{T,P,m_{j\neq i}}$$

if a solute concentration is low, ν_*i*_ can be used to express the solution volume change by a solute mass as

5 $$\Delta V\approx \nu_{i}m_{i}=\nu_{i}\left(c_{i}V\right)$$

where *c*_*i*_ is the mass concentration of the *i*-th solute (= *m*_*i*_/*V*). The relative volume change of a solvent can be expressed as (Δ*V*/*V*) = ν_*i*_ *c*_*i*_. In the noninteracting solute model, the relative volume change of a solvent in a solution must be proportional to *A*_solv_^excl^ as

6 $$A_{solv}^{excl}=\left(\frac{\Delta V}{V}\right)A_{solv}^{r}=\nu_{i}c_{i}A_{solv}^{r}$$

Then, eq 3 can be rewritten as

7 $$A_{solute}=A^{apparent}+A_{solv}^{excl}=A^{apparent}+\nu_{i}c_{i}A_{solv}^{r}$$

Thus, if we know ν_*i*_, *c*_*i*_, and *A*_solv_^r^, we can calculate *A*_solute_ from observed *A* ^apparent^. First, *c*_*i*_ can be either measured before dissolving a solute or estimated with other optical methods, such as UV–vis for protein and nucleic acid. *A*_solv_^r^ can be directly measured, as shown in Figure 2b, where the absorption spectrum of neat water was acquired by measuring the transmission spectra through an optical cell with different thicknesses (see the Supporting Information for details). Next, ν_*i*_ of common solutes can be found refs 15 and 16 or measured by a densitometry method, where the densities of solutions are measured with different solute concentrations by the following relation derived from the definition eq 4,^17^

8 $$\nu_{i}\approx \left(\frac{\Delta V}{m_{i}}\right)=\frac{1}{\rho_{solv}}\left(1-\frac{\rho_{soln}-\rho_{solv}}{c_{i}}\right)$$

where ρ_soln_ and ρ_solv_ are the densities of a solution and a solvent, respectively. When ρ_soln_, ρ_solv_, and *c*_*i*_ are known, one can determine ν_*i*_ with eq 8. The ν_*i*_ values of globular proteins range narrowly between 0.70 mL/g and 0.75 mL/g in water,^18,19^ although the ν_*i*_ values vary depending on temperature, pH, and the existence of other excipients (e.g., salts).

Before we perform the SE correction of *A* ^apparent^, we examine the concentration dependence of *A* ^apparent^. In eq 3, the first term *A*_solute_ can be easily replaced with ε_*i*_ *lc*_*i*_, and the second term *A*_solv_^excl^ can be expressed with *c*_*i*_ as *A*_solv_^excl^ = ν_*i*_ *c*_*i*_(*A*_solv_^r^) = ν_*i*_ *c*_*i*_(ε_solv_ *lc*_solv_^r^) from eq 6. Then, eq 3 becomes

9 $$A^{apparent}=\left(\varepsilon_{i}lc_{i}\right)-\nu_{i}c_{i}\left(\varepsilon_{solv}lc_{solv}^{r}\right)=\left(\varepsilon_{i}-\nu_{i}\varepsilon_{solv}c_{solv}^{r}\right)lc_{i}=\varepsilon_{i}^{\prime }lc_{i}$$

where the apparent absorption cross section, ε_*i*_^′^ = (ε_*i*_ – ν_*i*_ε_solv_ *c*_solv_^r^). We note that ε_*i*_^′^ is insensitive to *c*_*i*_, which enables *A* ^apparent^ to be still useful as a fingerprint of a specific solute–solvent system, although the SE effect modifies the spectral shape. Equation 9 also shows that *A* ^apparent^ is still proportional to *c*_*i*_, suitable for quantitative analysis of a solute or a multisolute mixture.

### SE Correction Using PSV for Protein Absorption Spectra

Figure 3 shows the raw spectra (*A* ^apparent^, the red solid lines) acquired from two globular proteins in aqueous solutions, which exhibit strong amide I and II peaks between 1500 cm^–1^ and 1700 cm^–1^. As described earlier in Figure 1a, negative absorbances were observed in the low-frequency region <1100 cm^–1^ and in the high-frequency region >1700 cm^–1^. For the calculation of *A*_solv_^excl^ in eq 6, *c*_*i*_ of each solution was measured with the UV-absorption at 280 nm; the separately measured water spectrum was used for *A*_solv_^r^ from Figure 2b; and the reported values of ν_*i*_ were used.^20^ The calculated *A*_solv_^excl^ were plotted as black dotted lines on the negative side of in Figure 3. Then, using eq 7, we calculated the SE-corrected solute spectra (*A*_solute_), which were plotted as blue solid lines in Figure 3.

**Figure 3:**
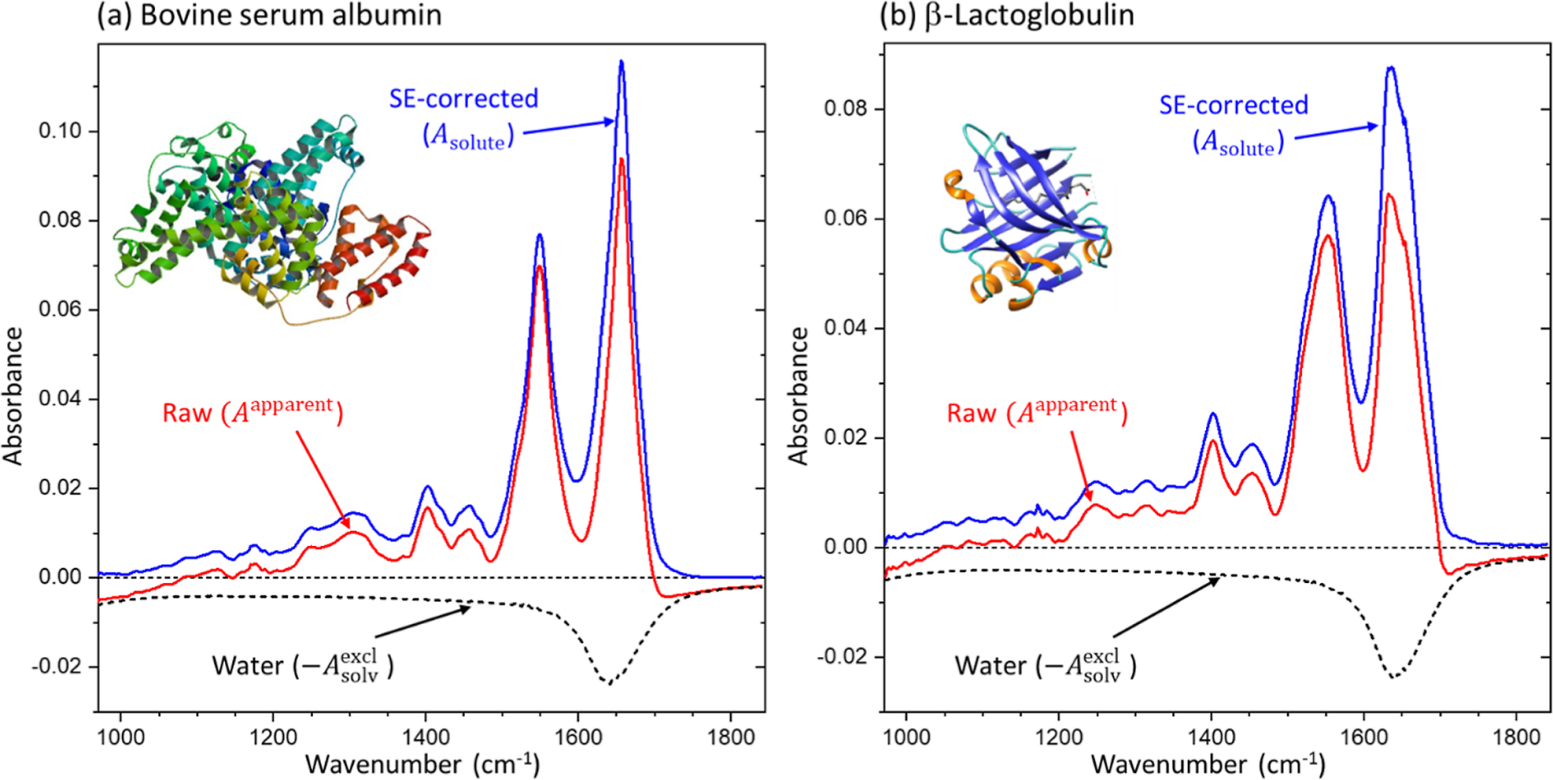
Correction of the SE effect of the absorption spectra acquired from (a) bovine serum albumin (BSA, 10 mg/mL, from Figure 1c) and (b) β-lactoglobulin (β-LG, 10 mg/mL) solutions. The SE effect is corrected with eq 7 using with the partial specific volumes (PSVs) of BSA and β-LG (ν_BSA_ = 0.737 mL/g and ν_βLG_ = 0.747 mL/g).^20^ The solid red lines are *A* ^apparent^ of the solutions, the dashed black lines are the spectrum of excluded water, *A*_solv_^excl^, and the solid blue lines are the absorption spectrum of the solutes, *A*_solute_.

We note that it is difficult to evaluate the performance of SE correction or the accuracy of a SE-corrected spectrum because there is no independent method to measure the solute-only spectrum from a solution, where the solute–solvent interaction is likely different from a powder or a crystal form. However, a simple guideline can be considered for protein. Typical proteins show negligible absorbance in the narrow window between 1740 cm^–1^ and 1840 cm^–1,^ while the absorption peak of the water bending mode is still non-negligible in the frequency range. The SE-corrected *A*_solute_ spectra show absorbance close to zero in the frequency region higher than 1740 cm^–1^, in Figure 3, indicating that the SE correction method using eq 7 works well with the independently measured *c*_*i*_, *A*_solv_^r^, and ν_*i*_ for the protein solutions. Figure S3 shows additional SE-corrected spectra of three other glycoproteins calculated with a common PSV of 0.373 mL/g.

Figure 3 demonstrated that the SE effect on an observed absorption spectrum was significant in an aqueous solution, particularly near the broad water absorption peak at 1644 cm^–1^. For the same reason, a SE-corrected absorption spectrum showed substantial deviation from the corresponding raw spectrum near 1644 cm^–1^, where the amide I band of protein happens to be located. The amide I band for a peptide unit is contributed by roughly 80% C=O stretching vibration, with the remaining contribution by out-of-phase C–N stretching and C–C–N deformation.^21^ The peak position of the amide I band shifts depending on the torsion angles along the backbone and the distance between O and N along linear C=O···H–N hydrogen bonds, which are susceptible to the secondary structure. Thus, the amide I band in IR is used to determine the secondary structure of a protein.^6,22^ For example, BSA, which is rich in α-helix (see its molecular structure in the inset of Figure 3a), shows a narrow amide I band peaked at 1655 cm^–1^, while β-LG, which is rich in β-sheet (see the molecular structure in the inset of Figure 3b), shows a broader peak at 1635 cm^–1^. Ideally, a protein’s amide I peak should be decomposable into underlying peaks corresponding to all existing secondary structures. However, in reality, the non-negligible heterogeneity in the hydrogen bonding strength, even of a single type of secondary structure, is difficult to predict due to variations in the peptide bond geometry of solutes and the configuration of interacting solvents,^23^ making it extremely difficult to determine the number of underlying peaks and their exact peak locations and widths. Thus, practically, we adopted a simple but widely used four-peak model for the amide I analysis studies to examine the effect of SE correction on the secondary structure analysis.^24^

Figure 4a–d show enlarged plots of the amide I peaks from the raw and SE-corrected spectra of the BSA and β-LG solutions in Figure 3. We subtracted the linear baseline of each spectrum before deconvoluting the amide I peak. We used four Gaussian functions for underlying peaks: α-helix centered at (1657 ± 2) cm^–1^; β-sheet(−) centered at (1638 ± 4) cm^–1^; β-sheet­(+) centered at (1685 ± 1) cm^–1^; and turns centered at (1672 ± 1) cm^–1^. We set all center positions and widths as common parameters, but with an allowance, as shown after the ± sign. β-sheet(−) and β-sheet­(+) indicate two antisymmetric stretching modes out of four eigenstates of the amide I transition dipole moments in β-sheet.^22^ Typically, the low-frequency β-sheet(−) peak is stronger than the high-frequency β-sheet­(+) peak. The four underlying peaks of each amide I peak are plotted in Figure 4a–d, and the relative areas of the underlying peaks are displayed as bar plots in Figure 4e,f. The center positions, the widths, and the relative areas are summarized with the peak assignments in Table 1. Overall, the SE correction increases the sum of β-sheet(−) and β-sheet­(+) more than α-helix, due to the asymmetricity of the water absorption spectrum with respect to the water bending peak (see Figure 2b). We note that the peak areas determined by the fitting do not accurately correspond to the true distribution of secondary structures. We must consider the fitting results as a coarse estimation or a simplified metric of relative structural change. For example, BSA is known to consist of α-helix dominantly, but the fitting results indicate the presence of about 60% or 70% of nonhelix structures. What we discussed earlier was the changes in the relative areas of α-helix and β-sheet after SE-correction.

**Figure 4:**
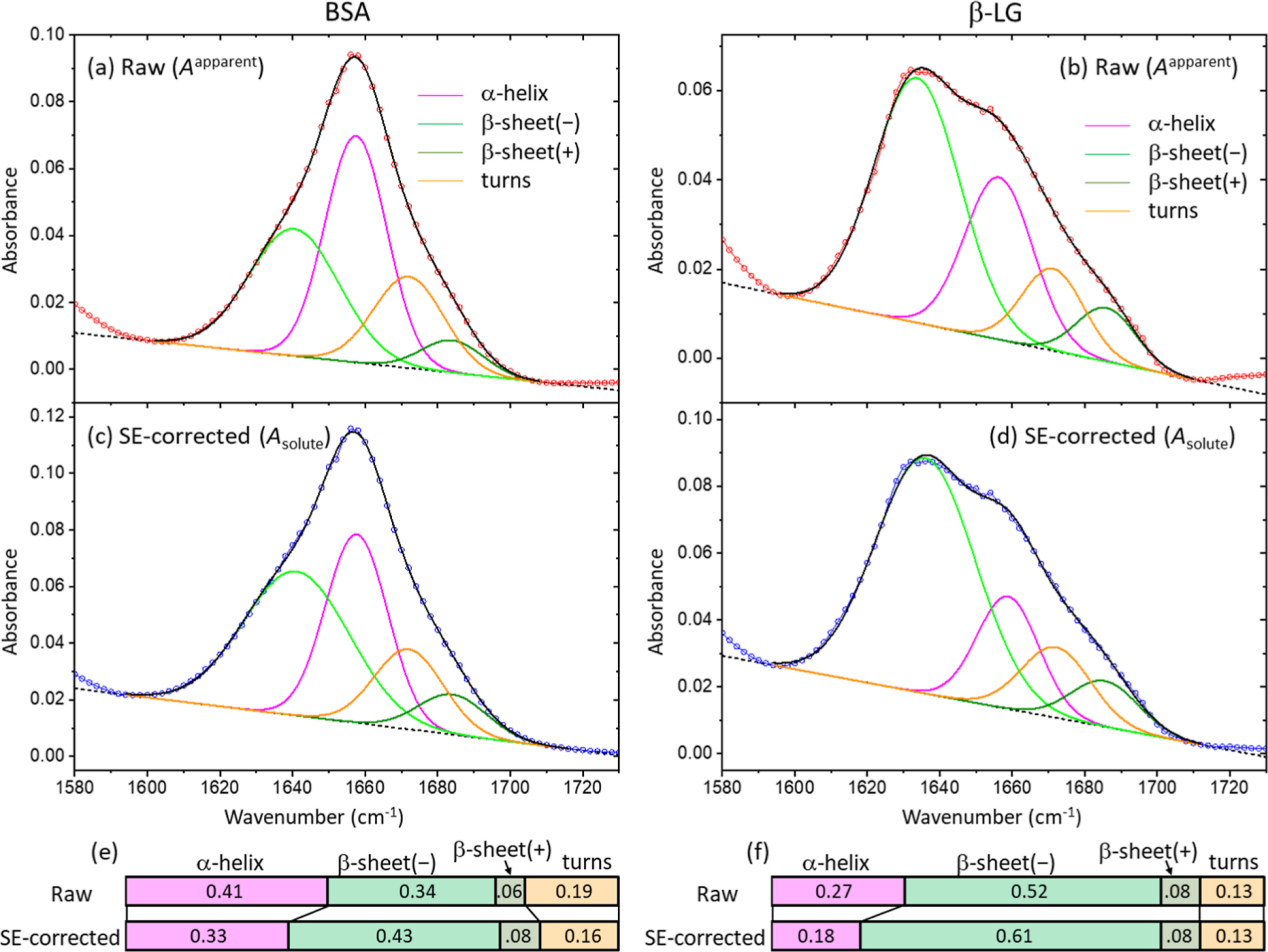
Comparisons of the composition analysis of the amide I peak of the raw and SE-corrected spectra of BSA and β-LG in Figure 3. (a–d) A peak was deconvoluted into four Gaussian functions with common parameters to all spectra. Before the fitting, each spectrum was subtracted by a line that touches the end of the amide I peak near 1600 cm^–1^ and 1710 cm^–1^. The center positions and widths of the four Gaussian functions were set as shared parameters with the allowance of ±3 cm^–1^ and ±2 cm^–1^, respectively. (e,f) Comparisons of the relative areas of the four underlying peaks calculated from the fitting of the raw and SE-corrected spectra.

**Table 1:** Summary of the Deconvolution Results of the Amide I Peak of the BSA and β-LG Solutions, Shown in Figure 4a–d^a^

		α-helix		β-sheet(−)		β-sheet(+)		turns	
		raw	SE-corrected	raw	SE-corrected	raw	SE-corrected	raw	SE-corrected
BSA	position (cm^–1^)	1657	1658	1640	1641	1684	1684	1672	1672
	width (cm^–1^)	17	17	25	29	17	19	19	19
	relative area	0.41	0.33	0.34	0.43	0.06	0.08	0.19	0.16
β–LG	position (cm^–1^)	1656	1659	1634	1636	1686	1686	1671	1672
	width (cm^–1^)	19	17	24	28	16	19	17	19
	relative area	0.27	0.18	0.52	0.61	0.08	0.08	0.13	0.13

a The positions of the four underlying peaks and the assignments of their corresponding secondary structures were adopted from ref 24.

### Change in a Solvent Spectrum due to an Interaction with a Solute

So far, we have explained how the SE effect can alter an IR absorption spectrum measured from an aqueous solution. Also, we have demonstrated a simple volumetric addition method to correct an apparent IR absorption spectrum to retrieve a solute-only absorption spectrum. The SE correction formula of eq 7 with PSV was derived based on the noninteracting solute model, which considers only the number of solvent molecules excluded by a solute. The simplified model does not consider a case when a solute interacts with solvating solvent molecules sufficiently strongly to change the IR absorption spectrum of the interacting solvent molecules. The simple volumetric correction based on eq 7 will fail: (1) when a solute–solvent interaction is sufficiently strong to change the absorption spectrum of solvent molecules in a solvation shell, and (2) when the number of the solvent molecules in a solvation shell is sufficiently greater than the number of excluded solvent molecules. It is difficult to quantitatively predict the solvent spectrum change caused by unknown solute–solvent interactions for the condition (1). However, we can test the condition (2) using a model to compare how many solvent molecules are excluded and how many solvent molecules are in the solvation shell as a function of solute size.

Figure 5a illustrates two cases for a model consisting of solute spheres (in solid red), solvent in bulk (in solid blue), and solvent in solvation shells (pattern-filled in blue/white). The volume of a core sphere increases with core diameter, and so does the volume of a solvation shell. However, their exponents are different; the core volume increases with the cube of core diameter, and the shell volume increases approximately with the square of core diameter. Figure 5b shows the calculation results of the volumes of a solute core and a solvation shell as a function of core diameter. To calculate the shell volume, we assumed the thickness of a solvation shell to be constant. The solvation shell thickness, more accurately, the correlation distance of a vibrational mode of solvent from a solute surface, can be inferred from various experimental and computational results. For example, the first water layer thickness from a mica–water interface was measured by X-ray reflectivity as 0.3 nm.^25^ The first water layer from a protein–water interface by molecular dynamics (MD) simulation was calculated to be 0.28 nm.^26^ Other more relevant estimations can be the radial correlation decays at 0.3 nm for water bending mode (≈1630 cm^–1^), 0.4 nm for collective hydrogen bond stretching vibrations (≈200 cm^–1^), and 0.6–0.7 nm for intermolecular vibrations (≈80 cm^–1^), determined by ab initio MD calculation and confirmed by terahertz spectroscopy.^27^ Thus, we used the thickness of 0.3 nm for a solvation shell. However, the solvation shell thickness may differ depending on various solution conditions, such as temperature, salt concentration, pH, etc.

**Figure 5:**
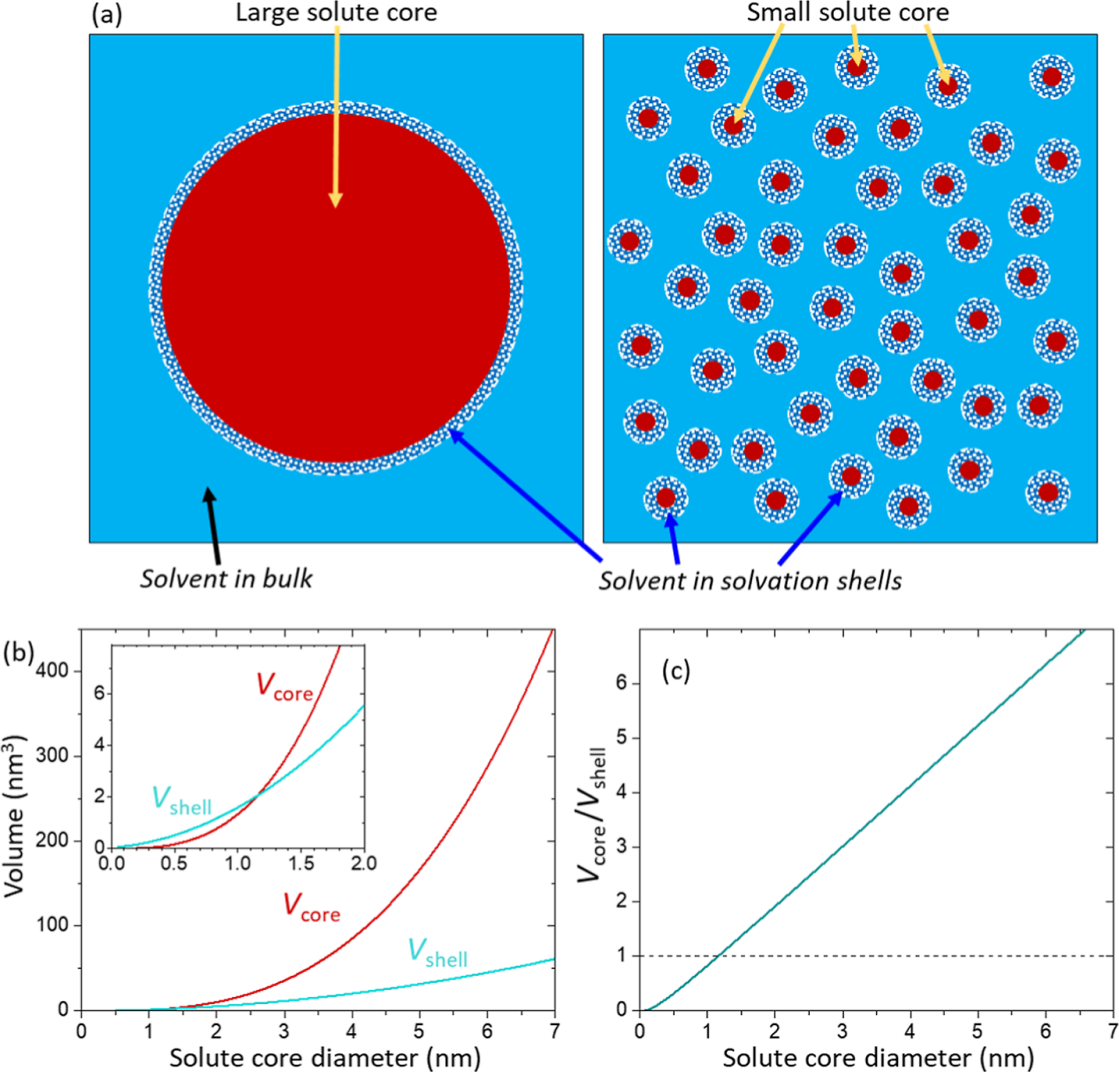
(a) Illustration of solutions consisting of solute cores (red), solvent in bulk (blue), and solvent in solvation shells (blue/shite). The thickness of solvation shells is assumed to be identical for solute cores with different sizes. (b) The core volume and the solvation shell volume were calculated as a function of core diameter. For the calculation of solvation shell volume, the solvation shell thickness was assumed to be constant at 0.3 nm (see the text). (c) Volume ratio of a core and a shell calculated as a function of core diameter.

In the inset of Figure 5b, the core volume is smaller than the shell volume for a core diameter smaller than 1.2 nm. However, when a core diameter is larger than 1.2 nm, the core volume becomes larger than the shell volume. The volume ratio of a core and a shell was plotted in Figure 5c as a function of solute diameter. The core–shell volume ratio corresponds to the number ratio of solvent molecules excluded by solute space and solute-interacting solvent molecules. This calculation indicates that when the solute size is large, the absorbance change by excluded solvent molecules overwhelms that by the solute–solvent interaction in the solvation shell. This calculation in Figure 5 must not be interpreted as a method to locate the exact crossing point, but instead, as a qualitative guideline to understand the difference in the effect of solute surface and volume with solute size.

The proteins studied in Figure 3 are both large globular proteins, which can be considered as the large solute core case in Figure 5a. The diameters of various globular proteins were calculated from the partial specific volume and the molecular weight. For example, 5.4 nm, for BSA; and 3.5 nm, for β-LG.^28^ A three-dimensional structure of BSA calculated from the protein database was (9 × 5.5 × 5.5 nm^3^) in volume.^29^ Experimentally, dynamic light scattering was used to measure the hydrodynamic diameter of BSA, which was about 4 nm.^30^ The diameter of a β-LG monomer was measured to be 3.6 nm by small-angle X-ray scattering,^31^ but depending on pH and salt concentration, β-LG can also exist in larger-sized dimer or tetramer forms.^32^ The globular shape and the large size of BSA and β-LG strongly suggest that the volumetric SE correction by eq 7 should be effective, and actually, the correction results in Figure 3 indicate that the SE correction with reported PSV values was successful.

Unlike a large globular protein solution, a small molecule solution is expected to have a more significant contribution from solvation shells. If the solvent molecules in a solvation shell have a different spectrum from the bulk solvent, the effect will be uncorrectable by the simple volumetric addition. For example, the hydrogen bond network of the water in the solvation shell can be perturbed by the presence of a polar or charged functional group in a solute molecule. The perturbation distance (≈the solvation shell thickness) and the resulting IR spectral change will vary depending on various parameters, including partial charge state, solution pH, codissolved salt, temperature, etc. We examined several aqueous solutions consisting of small molecule solutes to see the performance of the volumetric SE correction with PSV by eq 7. Among them, we present two examples of different solute–solvent interactions: a mannose solution and a spermine solution from Figure 1b,c, respectively. Mannose consists of five hydroxyl groups (−OH), which can interact with neighboring water via hydrogen bonding. On the other hand, spermine’s four amine groups, positively charged at pH 7, might have perturbed the water hydrogen bonding network more significantly than neutral mannose.

First, Figure 6a shows the raw absorption spectra of the mannose solution, which was converted into an *A*_solute_ spectrum using ν_*i*_ = 0.62 mL/g,^16^ in Figure 6c. After the SE correction, a distinct peak remains near the water bending mode at 1645 cm^–1^. Interestingly, the peak must not be from a mannose molecule without water from the absence of a peak near 1645 cm^–1^ because the IR spectrum of mannose solid crystals shows no peak (see Figure S1).^33^ This suggests that the 1645 cm^–1^ peak in the *A*_solute_ spectrum is unlikely from mannose molecular vibrations but may be due to the spectral change of the water. To check if the positive peak is due to inaccurate inputs of ν_*i*_ or other input parameters, such as *c*_*i*_, we also performed the SE correction with two other ν_*i*_ values, one above and the other below the reported ν_*i*_ value. In all SE-corrected *A*_solute_ spectra, shown in Figure 6c, the 1645 cm^–1^ peak remains. When a smaller value of ν_*i*_ was used to lower the 1645 cm^–1^ peak height, the high-frequency side of the peak became asymmetrically negative, as shown in the plot calculated with ν_*i*_ = 0.52 mL/g. The existence of the uncorrectable peak at the water bending frequency suggests that the SE correction cannot be completed for the mannose solution only with the volumetric subtraction with a PSV value. We have examined a different monosaccharide to confirm if other solute molecules could have a similar feature to mannose. A raw absorption spectrum of a galactose solution was SE corrected with ν_*i*_ = 0.62 mL/g, as shown in Figure S2. The output *A*_solute_ spectrum of the galactose solution showed a similar peak at 1645 cm^–1^ to that of the mannose solution, indicating the spectral changes and the solvation shell volume are similar between the two monosaccharides.

**Figure 6:**
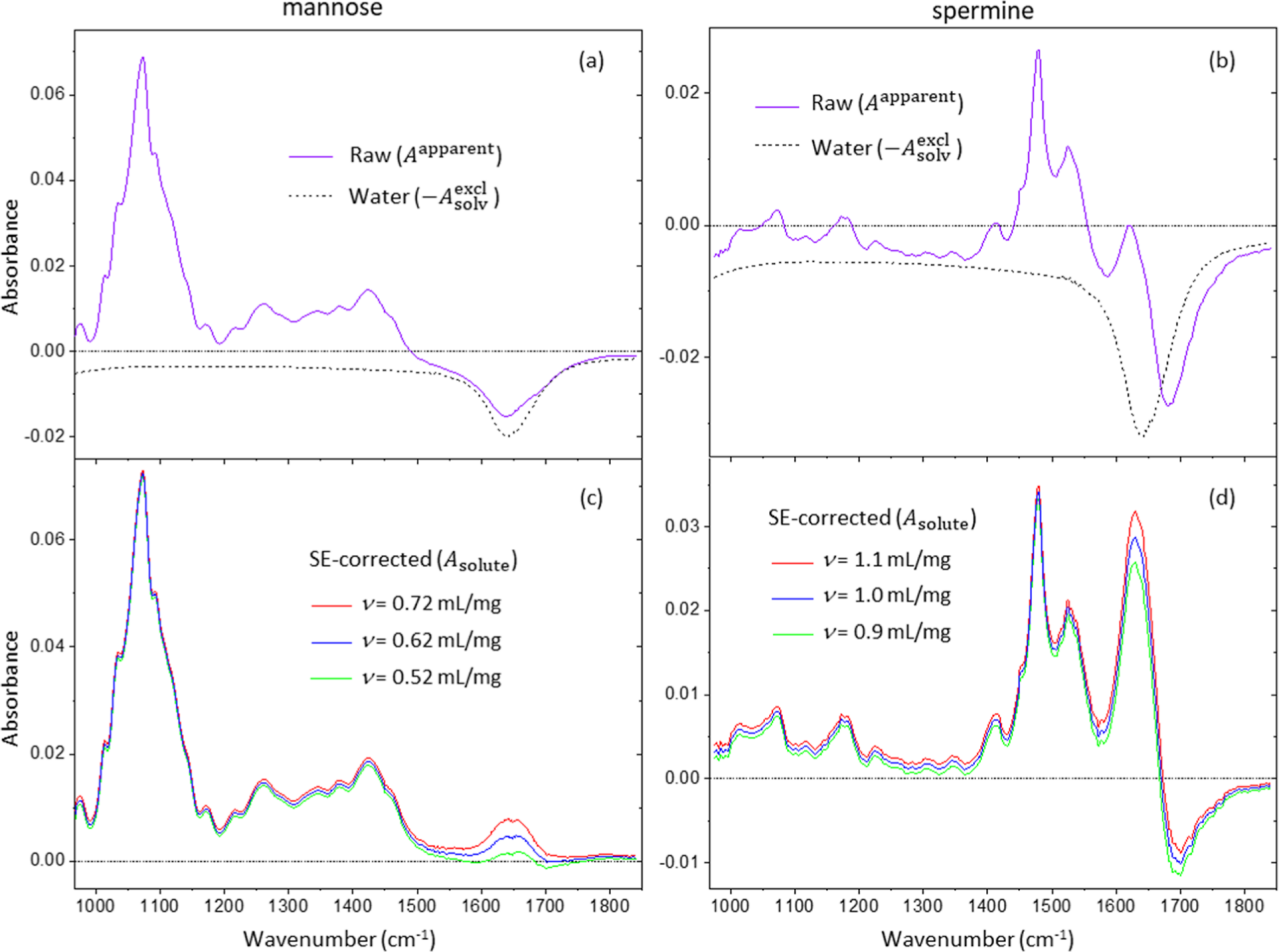
SE correction results of the absorption spectra of mannose and spermine solutions from Figure 1b,c. (a) Raw spectrum of the mannose solution compared with the excluded water spectrum calculated using ν_*i*_ = 0.62 mL/g.^16^ (b) Raw spectrum of spermine solution compared with the excluded water spectrum calculated using ν_*i*_ = 1.0 mL/g (see the text). (c) SE-corrected spectra calculated with three different PSV values (ν_*i*_ ± 0.1 mL/mg) for mannose. (d) SE-corrected spectra calculated with three different PSV values (ν_*i*_ ± 0.1 mL/mg) for spermine.

Second, the PSV value of spermine was not available, but the PSV values of similar linear polyamines were reported, ranging from 0.93 mL/mg to 1.07 mL/g.^15^ Thus, we used three ν_*i*_ values of 0.9 mL/g, 1.0 mL/g, and 1.1 mL/g, covering the range of reported linear polyamines to calculate the SE-corrected *A*_solute_ spectrum of the spermine solution. The SE-corrected spectra, shown in Figure 6d, display a large negative absorbance around 1700 cm^–1^. The distortion of the spermine solution is much more significant than that of the mannose solution. A difference between the two samples is the charge state. The high p*K*_a_ values (7.9, 8.4, 10.1, and 10.9) of spermine will make all four amine groups positively charged. We suspect that the highly charged state of a small-molecule polyamine perturbed the solvent hydrogen bond network significantly and caused the corresponding spectral distortion to the solvent in the solvation shell.

In short, while the apparent absorption spectrum of a solution containing large globular solutes is dominantly contributed by volumetric SE, the apparent absorption spectrum of a solution with small solutes can additionally be affected by spectral changes of surrounding solvents. Thus, when we analyze absorption spectra of solutions containing small-molecule excipients, e.g., bioprocessing samples and biological drug products, we must consider the potential effect of spectral changes of solvent molecules strongly interacting with solutes, such as salts (see Figure S4). Thus, for an accurate absorption measurement, the solution formulations must be maintained as close as possible between a sample and a reference, except for the analyte.

So far, we have focused the discussion on the SE effect to dilute homogeneous solutions. However, we need to be cautious when analyzing a sample prepared in different conditions. For example, when a solute concentration is as high as solute molecules feel each other, PSV will no longer be a constant value, and the shape of the solute’s spectrum can also change due to the proximity. Also, the refractive index of a high-concentration solution can be different from that of a dilute solution, and the resulting difference in reflectivity of the sample cell interfaces will alter the apparent absorbance. Furthermore, if a solute or a solvent is highly absorbing, their dispersion effect and local field effect will make the transmission vary with wavelength, causing the apparent absorption spectrum to become nonlinear with solute concentration.^34,35^ When a solute exists in the form of aggregates or microparticles, the scattering can alter not only the spectral shape but also the concentration linearity.

## Conclusion

Based on the QCL-IR absorption spectroscopy, we examined the SE effect on an apparent absorbance spectrum from aqueous solutions. We derived a simple subtractive correction method based on PSV to retrieve a solute-only absorption spectrum. The mathematical method using a simple subtractive formula with previously reported PSV values effectively recovered the absorption spectra of globular proteins. We also demonstrated that the SE effect on the absorption spectra of solutions with small-molecule solutes could not be fully explained by the PSV-based subtractive formula. We discussed that the potential origins of the non-negligible spectral deviation might be due to a relatively significant contribution of solvents in the solvation shell compared to the excluded solvent volume. When a solute perturbs the hydrogen bonding network of solvents more strongly, the change in the solvent spectrum will result in a greater effect that cannot be corrected by the volumetric subtractive formula. On the other hand, further comparative studies on the volumetric and nonvolumetric SE effects will facilitate the understanding of the solute–solvent interaction and its effect on the solvent spectrum in the solvation shell.

## Supplementary Material



## Notes

Measurement procedure of the absorption spectrum of neat water; IR spectra of mannose observed in an aqueous solution and a solid; SE correction of the absorption spectrum of a galactose solution; SE correction of three glycoprotein spectra; and absorption spectrum of a 0.15 M NaCl aqueous solution (PDF)

## Notes

DISCLAIMER Certain commercial equipment, instruments, or materials are identified in this paper to foster understanding. Such identification does not imply endorsement by NIST, nor does it imply that the materials or equipment identified are necessarily the best available for the purpose. Official contribution of the National Institute of Standards and Technology; not subject to copyright in the United States.
