# Supplementary material for: Solvent Exclusion Effect on Infrared Absorption Spectroscopy
Source: Anal Chem. 2025 Jul 9;97(28):15453–62. doi: 10.1021/acs.analchem.5c02700 (PMC12291039; doi:10.1021/acs.analchem.5c02700)

Supplementary Information

Solvent Exclusion Effect on Infrared Absorption Spectroscopy

Young Jong Lee[1,*], *Seong-Min Kim*[1], *Sang Hak Lee*[1,2], *Charles Camp Jr.*[1], *Bonghwan Chon*[1]

[1] Biosystems and Biomaterials Division, National Institute of Standards and Technology, Gaithersburg, Maryland 20899, USA

[2] Department of Chemistry, Pusan National University, Busan, 46241, Korea

* E-mail: youngjong.lee@nist.gov

Measurement of the absorption spectrum of neat water

Transmission spectra were measured consecutively with two different thickness spacers (the nominal thicknesses of 15 μm and 25 μm) in a detachable cell filled with neat water. The SAC was recalibrated for a thickness of 15 μm. The transmission through the 15 μm thick cell was used as a reference, and the transmission through the 25 μm was used as a sample. This thickness-varying scheme removes the common artifacts that can affect the transmission of water, such as the reflection from the interfaces of air/window and window/water. The inaccuracy in the water path length calculated from the nominal thickness was calibrated with the reference absorption coefficient of liquid water at 20 °C.

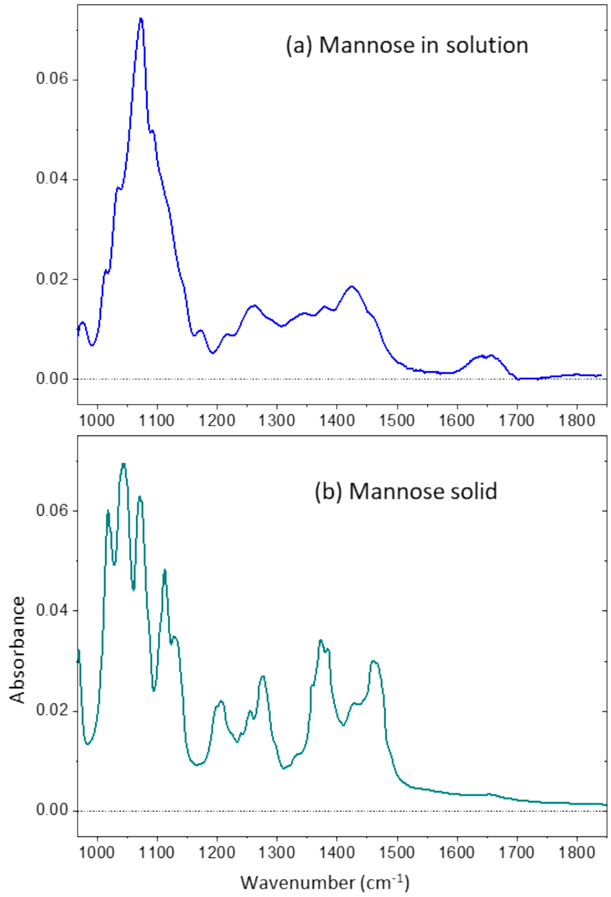

Figure S1. (a) SE-corrected IR spectrum of mannose in an aqueous solution with a partial specific volume of ν_i = 0.62 mL/g. (b) IR spectrum of a solid [Stein, S. E. IR and Mass Spectra. In *NIST Chemistry WebBook*, Mallard, W. G., Linstrom, P. J. Eds.; NIST Standard Reference Database Number 69, National Institute of Standards and Technology, 2000].

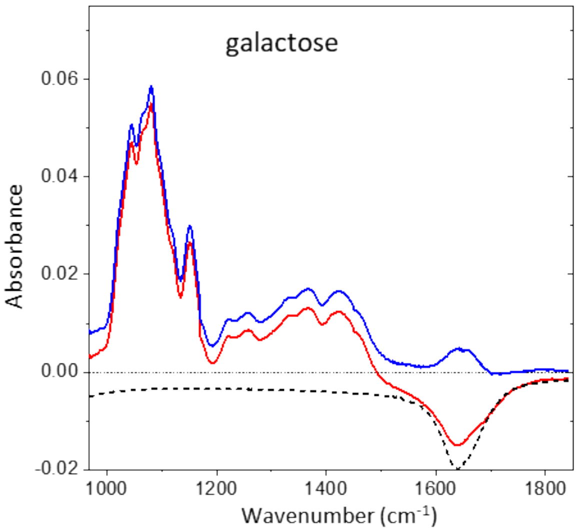

Figure S2. SE-correction of an absorption spectrum of galactose in an aqueous solution (10 mg/mL). The red solid lines are A^{apparent} of the solutions, the black dashed lines are the spectrum of excluded water, $A_{\mathrm{solv}}^{\mathrm{excl}}$, and the blue solid lines are the absorption spectrum of the solutes, A_{solute}, after the solvent exclusion effect is removed with Eq. **Error! Reference source not found.** using ν_i = 0.62 mL/g.

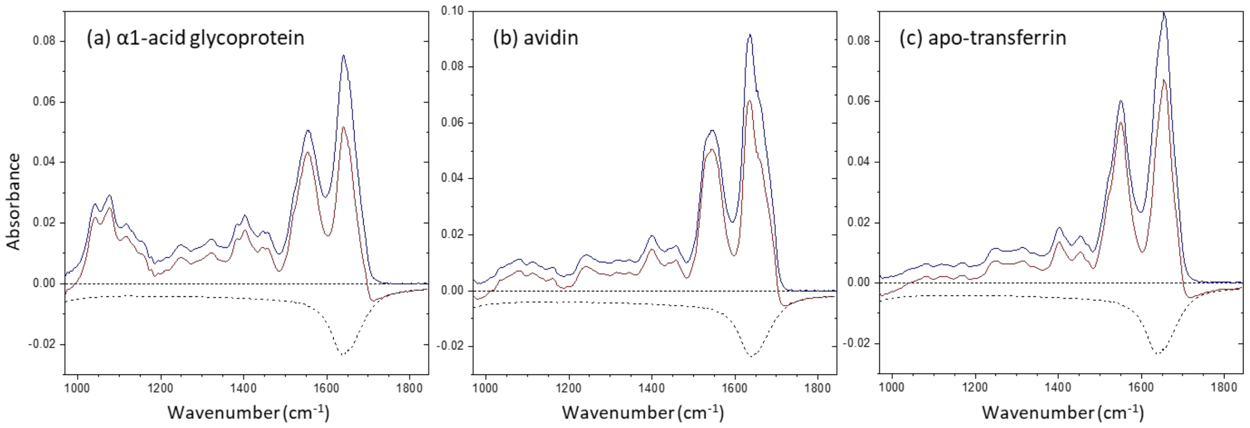

Figure S3. Solvent exclusion correction of the absorption spectra of three glycoprotein solutions (from Kim et al. *Anal. Chem.* 96, 13120 (2024)). A common PSV value of 0.737 mL/g was used for the SE correction for all solutions. The red lines represent the apparent absorbance spectra, and the blue lines represent the SE-corrected spectra. The black dotted lines are the absorption of water with a path length of 26 μm.

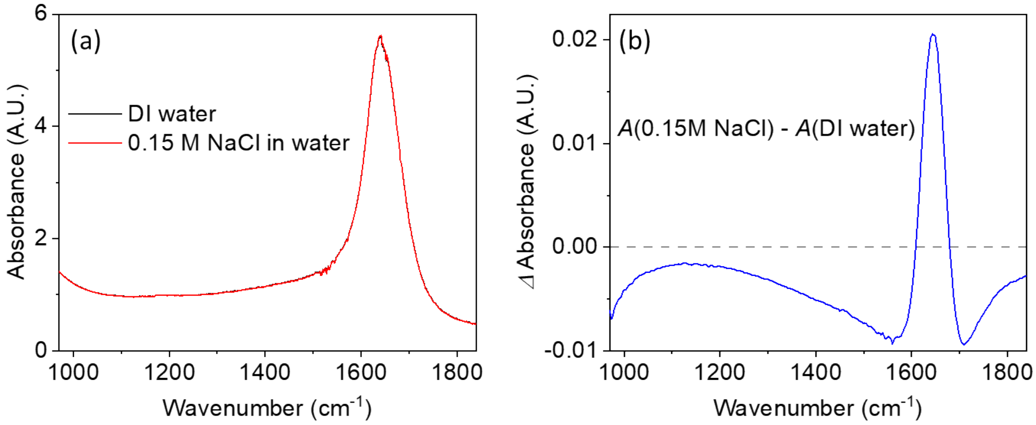

Figure S4. (a) Absorption spectra of deionized (DI) water and a 0.15 M NaCl solution through a path length of 26 μm. (b) Absorbance difference spectrum between the 0.;15 M NaCl solution and DI water.

Table of Content Figure

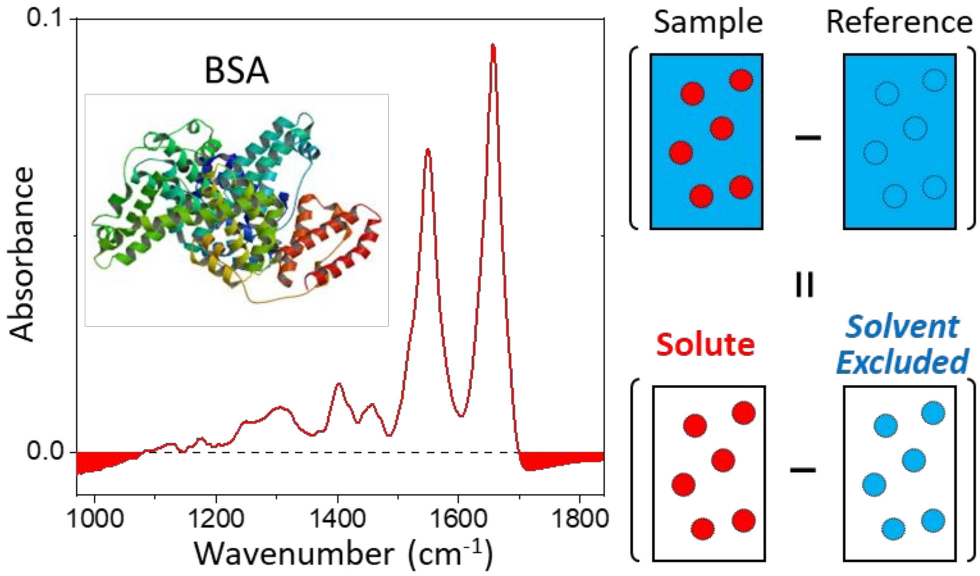

Supplement: Supplementary file 1 [file ac5c02700_si_001.pdf]
